# Strain-specific bioaccumulation and intracellular distribution of Cd^2+^ in bacteria isolated from the rhizosphere, ectomycorrhizae, and fruitbodies of ectomycorrhizal fungi

**DOI:** 10.1007/s11356-014-3489-0

**Published:** 2014-09-19

**Authors:** Katarzyna Hrynkiewicz, Michał Złoch, Tomasz Kowalkowski, Christel Baum, Katarzyna Niedojadło, Bogusław Buszewski

**Affiliations:** 1 Department of Microbiology, Faculty of Biology and Environmental Protection, Nicolaus Copernicus University, Lwowska 1, 89-100 Torun, Poland; 2 Chair of Environmental Chemistry and Bioanalytics, Nicolaus Copernicus University, Gagarina 7, 87-100 Torun, Poland; 3 Department of Agricultural and Environmental Sciences, Soil Science, University of Rostock, Justus-von-Liebig-Weg 6, 18059 Rostock, Germany; 4 Department of Cell Biology, Institute of General and Molecular Biology, N. Copernicus University of Torun, Torun, Poland

**Keywords:** Cadmium, Bioaccumulation, Rhizosphere bacteria, Transmission electron microscopy, X-ray microanalysis

## Abstract

Bioaccumulation of Cd^2+^ in soil bacteria might represent an important route of metal transfer to associated mycorrhizal fungi and plants and may have potential as a tool to accelerate Cd^2+^ extraction in the bioremediation of contaminated soils. The present study examined the bioaccumulation of Cd^2+^ in 15 bacterial strains representing three phyla (*Firmicutes*, *Proteobacteria*, and *Bacteroidetes*) that were isolated from the rhizosphere, ectomycorrhizae, and fruitbody of ectomycorrhizal fungi. The strains *Pseudomonas* sp. IV-111-14, *Variovorax* sp. ML3-12, and *Luteibacter* sp. II-116-7 displayed the highest biomass productivity at the highest tested Cd^2+^ concentration (2 mM). Microscopic analysis of the cellular Cd distribution revealed intracellular accumulation by strains *Massilia* sp. III–116-18, *Pseudomonas* sp. IV-111-14, and *Bacillus* sp. ML1-2. The quantities of Cd measured in the interior of the cells ranged from 0.87 to 1.31 weight % Cd. Strains originating from the rhizosphere exhibited higher Cd^2+^ accumulation efficiencies than strains from ectomycorrhizal roots or fruitbodies. The high Cd tolerances of *Pseudomonas* sp. IV-111-16 and *Bacillus* sp. ML1-2 were attributed to the binding of Cd^2+^ as cadmium phosphate. Furthermore, silicate binding of Cd^2+^ by *Bacillus* sp. ML1-2 was observed. The tolerance of *Massilia* sp. III-116-18 to Cd stress was attributed to a simultaneous increase in K^+^ uptake in the presence of Cd^2+^ ions. We conclude that highly Cd-tolerant and Cd-accumulating bacterial strains from the genera *Massilia* sp., *Pseudomonas* sp., and *Bacillus* sp. might offer a suitable tool to improve the bioremediation efficiency of contaminated soils.

**Electronic supplementary material:**

The online version of this article (doi:10.1007/s11356-014-3489-0) contains supplementary material, which is available to authorized users.

## Introduction

Cadmium (Cd^2+^) is not involved in any known biological processes and is one of the most critical heavy metal pollutants due to its high solubility in water (Pinto et al. 2003), rapid accumulation in the food chain, severe toxic effects on living organisms, and mutagenic nature (Rani and Goel 2009; Farooq et al. 2010). Cd^2+^ exposure has numerous effects in humans, including kidney damage, high blood pressure, and bone fractures (Drasch 1983), and is acknowledged as a priority pollutant by the US Environmental Protection Agency (USEPA) (Krishnan and Anirudhan 2003). The European Chemicals Agency (ECHA) has placed Cd^2+^ on the Candidate List of Substances of Very High Concern for Authorisation (SVHC), and its content in products is subjected to rigorous restrictions [Regulation (EC) No. 1907/2006 of the European Parliament and of the Council, 2006]. Cd is commonly present in wastes from technological processes such as plastic manufacturing, galvanizing plating or pigments, and Cd/Ni battery production. Cd^2+^ is highly toxic in even relatively low concentrations; the World Health Organization (WHO) describes the highest permissible level of this element as 0.003 mg L^−1^ (Cadmium in drinking water 2011). Cd^2+^ soil contamination can decrease crop yields by inhibiting photosynthesis, respiration, and nitrogen metabolism and by reducing water and mineral uptake (Tynecka et al. 1981). Microorganisms associated with plant roots can increase biomass production at sites polluted with heavy metals, affect metal mobilization in the soil, and protect the host plant from heavy metal toxicity (Hrynkiewicz and Baum 2012; 2013).

Many bacteria are tolerant to high concentrations of Cd^2+^; however, this element generally causes reduced growth, long log phases, lower cell densities, and even bacterial death (Les and Walker 1984; Sinha and Mukherjee 2009). The presence of Cd^2+^ in the environment can affect the survival and composition of bacterial populations, thereby negatively affecting plant growth (Siripornadulsil and Siripornadulsil 2013) and impacting the entire ecosystem. Bacteria have developed several mechanisms for the detoxification of Cd^2+^, e.g., extracellular and intracellular sequestration (Hetzer et al. 2006), active efflux out of the cell (Teitzel and Parsek 2003), and biotransformation into less toxic forms (Khan et al. 2010), which allow these bacteria to tolerate increased concentrations of Cd^2+^ ions in the environment (Nies 1999).

A fundamental strategy to avoid the toxic effects of Cd^2+^ ions that is widespread among bacterial species is to prevent the ions from entering the cell and to keep them away from the target sites (exclusion). This process may involve three different mechanisms: adsorption on the cell surface, secretion of high amounts of viscous slime outside the cells, and/or precipitation (Bruins et al. 2000; Zamil et al. 2008; Deb et al. 2013). The bacterial cell wall is the first component that interacts with heavy metal ions (Yun et al. 2011). Mechanisms of cell surface sorption are passive and based upon physicochemical interactions between the metal and the functional groups of the cell wall; this process occurs independently of cell metabolism (Oh et al. 2009). The negatively charged bacterial cell wall can bind high quantities of positively charged Cd^2+^ ions, thereby immobilizing the metal and inhibiting its intracellular toxic effects (Goulhen et al. 2006; Parungao et al. 2007). The carboxyl groups responsible for forming Cd-carboxyl complexes on the bacterial surface may be involved in this process (Yee and Fein 2001). Moreover, some bacteria secrete exopolysaccharides or capsular materials that bind high amounts of Cd^2+^ (Ron et al. 1992). Exopolysaccharides synthesized by *Arthrobacter viscosus* accumulate 2.3 times more Cd^2+^ than an equivalent weight of intact cells and have 13.7 times the sorptive capacity of *Arthrobacter globiformis* cells, which do not produce exopolysaccharides (Scott and Palmer 1988). This phenomenon of Cd^2+^ ion accumulation in capsular material has been observed in *A. viscosus* and *Klebsiella aerogenes* (Scott and Palmer 1988, Scott and Palmer 1990). Cd^2+^ influx into bacterial cells can also be limited by external precipitation (Bruins et al. 2000). *Citrobacter* sp. can resist Cd^2+^ toxicity by forming insoluble complexes of Cd-phosphate (McEntee et al. 1986), while *K. aerogenes* excretes sulfur into the surrounding environment to immobilize Cd^2+^ ions as insoluble Cd-sulfide (Aiking et al. 1982; Scott and Palmer 1990).

Increased bacterial tolerance to Cd stress is accomplished through the intracellular immobilization of ions accumulated in bacterial cells. Magnesium (Gram-negative) or manganese (Gram-positive) uptake systems sequester Cd^2+^ by internal precipitation or by binding Cd^2+^ to thiol-rich peptides (Ron et al. 1992; Nies 1995). One *Citrobacter* mutant isolated from metal-polluted soil accumulates Cd^2+^ as insoluble cell-bound CdHPO_4_; this transformation is mediated by a cell-bound phosphatase that precipitates inorganic phosphate with heavy metals (Macaskie et al. 1987). A strain of *Pseudomonas putida* isolated from sewage can sequester intracellular Cd^2+^ by producing three low-molecular-weight cysteine-rich proteins related to eukaryotic metallothioneins (Trevors et al. 1986). Moreover, some bacterial antioxidant defense enzymes, e.g., catalase, peroxidase, and superoxide dismutase (SOD), can participate in monitoring metal ion levels and respond accordingly by regulating gene expression (Horsburgh et al. 2001).

Bacterial detoxification of Cd^2+^ can also be accomplished by active efflux of the ions (Tynecka et al. 1981). In Gram-negative bacteria, Cd^2+^ appears to be detoxified by RND-driven systems such as Czc, which is mainly a zinc exporter (Nies 1995; Nies and Silver 1989), and Ncc, which is mainly a nickel exporter (Nies 1995; Schmidt and Schlegel 1994). The Czc system is driven by a H^+^ ion gradient that allows CzcA (located in the inner membrane) to pump Cd^2+^ out of the cytoplasm (Diels et al. 1995). The presence of this system has been confirmed, inter alia, for *Ralstonia metallidurans* and *Ralstonia eutropha* (Nies 1995; Rani and Goel 2009). In Gram-positive species, the removal of Cd^2+^ from the interior of the cell is mediated by the CadA pump, which acts as a Cd-exporting P-type ATPase; this mechanism has also been described for *Staphylococcus aureus* (Nucifora et al. 1989; Silver et al. 1989). The presence of CadA-like proteins has also been confirmed in other Gram-positive bacteria, such as *Bacillus* sp. and *Listeria* sp. (Bruins et al. 2000). Although detoxification of Cd^2+^ by biological methylation has been proposed, there is no conclusive evidence for microbial transformation of Cd^2+^ (Ron et al. 1992).

Several studies have confirmed the ability of heavy-metal-resistant bacteria to accumulate Cd^2+^ in their living cells (Malik 2004). Roane and Pepper (2000) observed that bacterial strains originating from metal-contaminated soils had different capacities to remove Cd^2+^ from growth media; these varying capacities were most likely due to differing Cd^2+^ resistance mechanisms. Surprisingly, the most efficient strains in this investigation comprised both Gram-negative (*Pseudomonas* strain H1) and Gram-positive (*Bacillus* strain H9) bacteria. Similarly, Haq et al. (1999) observed a significant reduction in the Cd^2+^ content in the medium during the growth of bacteria derived from industrial effluents but did not observe strain-specific differences in the mechanism of resistance. These data suggest that the use of growing microbial cells could be a very promising method of Cd^2+^ removal that, unlike conventional biosorption techniques, exhibits a constant capacity. However, a more accurate overview of the mechanisms of Cd^2+^ accumulation at the cellular level is needed (Roane and Pepper 2000).

The main objectives of this study were (i) to select the most effective Cd-accumulating bacterial strains from 15 bacterial strains isolated from the rhizosphere, ectomycorrhizae, and fruitbody of ectomycorrhizal fungi; (ii) to determine the strain-specific distribution of absorbed Cd^2+^ within single cells; and (iii) to assess atomic absorption spectrometry (AAS) as a suitable tool to indicate the Cd^2+^ uptake capacity of bacteria for future bioremediation application.

## Materials and methods

### Bacterial strains

A total of 15 bacterial strains were isolated from the rhizosphere, ectomycorrhizae, and fruitbodies of ectomycorrhizal fungi associated with willows (*Salix viminalis* L.) growing at anthropogenic degraded sites. The examined strains represented three phyla (*Firmicutes*, *Proteobacteria*, and *Bacteroidetes*) and were characterized by different cell wall compositions, both Gram-negative and Gram-positive. Four strains from the class *Bacilli* (*Bacillus cereus* B1, B2, B3, B4) had been previously identified and described by Hrynkiewicz et al. (2012). Six strains from the class *Gammaproteobacteria* (*Pseudomonas* sp. IV-111-14, *Luteibacter* sp. II-116-7, *Serratia entomophila* I-111-21), *Betaproteobacteria* (*Massilia* sp. III-116-18), *Bacteroidetes* sp. I-116-1 and *Flavobacterium* sp. I-111-11 were selected from a group of 50 investigated bacterial strains on the basis of physiological properties by Hrynkiewicz et al. (2010). The strain *Pseudomonas fulva* was isolated from the interior tissues of the roots of Scots pine trees growing in sand dunes at the Baltic Sea of Poland (Strzelczyk and Li 2000), a noncontaminated site. The remaining four strains, *Bacillus* sp. ML1-2, *Luteibacter rhizovicinus* ML3-1, *Variovorax* sp. ML3-12, and *Pseudomonas fluorescens* LIC 1, were isolated from ectomycorrhizae or fruitbodies collected at heav- metal-contaminated test sites (Hrynkiewicz et al. 2008) and identified and described for the first time (based on 16S rDNA) in this work. The identification procedure was performed according to Hrynkiewicz et al. (2010, 2012) with small modifications. The DNA of bacterial strains was isolated using Dneasy® Blood & Tissue Kit (Qiagen, Hilden, Germany). The amplification of the 16S ribosomal RNA (16S rRNA) gene was performed using Taq PCR Master Mix (Qiagen, Hilden, Germany) and primers F1 (5′-GAG TTT GAT CCT GGC TCA G-3′) and R12 (5′-ACG GCT ACC TTG TTA CGA CTT–3′) (Dorsch and Stackebrandt 1992). The thermal program comprised an initial denaturation step of 2 min at 95 °C, followed by 30 cycles of 1-min denaturation at 94 °C, 1-min annealing at 55 °C, and 2-min extension at 72 °C, and a final elongation step of 5 min at 72 °C. The PCR products were purified using the QIAquick PCR Purification Kit (Qiagen, Hilden, Germany). Direct sequencing of PCR products was performed using the PCR primers as sequencing primers. Sequences were aligned manually aided by the Sequencher system (TW Version 5.1, Gene Codes, Ann Arbor, MI, USA). The BLAST database (Altschul et al. 1997) of the National Center for Biotechnology Information (NCBI) was used to compare obtained sequences of all isolates with known 16S rRNA gene sequences of bacterial strains. The DNA sequences determined in this study were deposited in GenBank under accession numbers [KM411501], [KM411502], [KM411503], [KM411504] (Table 1). The results of molecular identification of newly identified bacterial strains were presented in the form of phylogenetic tree and presented in the supplementary data (Fig. 4).

**Table 1:** Bacterial strains used in the study

Nr	Strain	Accession number	Origin^a^	References
Phylum *Firmicutes*, class *Bacilli*				
1	*Bacillus cereus* B1	HM989918	M	Hrynkiewicz et al. (2012)
2	*Bacillus cereus* B2	HM989916	M	Hrynkiewicz et al. (2012)
3	*Bacillus cereus* B3	HM989917	M	Hrynkiewicz et al. (2012)
4	*Bacillus cereus* B4	HM989919	F	Hrynkiewicz et al. (2012)
5	*Bacillus* sp. ML1-2	[KM41111502]^a^	M	This work
Phylum *Proteobacteria*, class *Gammaproteobacteria*				
6	*Pseudomonas fluorescens* LIC 1	[KM411503]^a^	F	This work
7	*Pseudomonas fulva*	[KM411501]^a^	R	This work
8	*Pseudomonas* sp. IV-111-14	FJ786066	R	Hrynkiewicz et al. (2010)
9	*Luteibacter rhizovicinus* ML3-1		M	This work
10	*Luteibacter* sp. II-116-7	FJ786052	R	Hrynkiewicz et al. (2010)
11	*Serratia entomophila* I-111-21	FJ786072	R	Hrynkiewicz et al. (2010)
Phylum *Proteobacteria*, class *Betaproteobacteria*				
12	*Massilia* sp. III-116-18	FJ786054	R	Hrynkiewicz et al. (2010)
13	*Variovorax* sp. ML3-12	[KM411504]^a^	M	This work
Phylum: *Bacteroidetes*				
14	*Bacteroidetes* sp. I-116-1	FJ786045	R	Hrynkiewicz et al. (2010)
15	*Flavobacterium* sp. I-111-11	FJ786048	R	Hrynkiewicz et al. (2010)

*M* mycorhizosphere, *F* fruitbody, *R* rhizosphere

^a^Accession numbers will be given

### Concentration of Cd in bacterial biomass—AAS analysis

Analyses of Cd^2+^ accumulation were performed using the biomass of 3-day bacterial cultures (liquid medium R2A, Difco, Franklin Lakes, NJ, USA) supplemented with CdSO_4_ × 7H_2_O to final Cd concentrations of 0.5, 1.0, 2.0, and 3.0 mM. The chemicals present in the medium can induce Cd^2+^ ion precipitation. To evaluate the real availability of soluble Cd^2+^ to the cultivated bacterial strains in the medium, the concentration of available Cd^2+^ ions was measured by AAS. The control samples were cultured in liquid medium without added heavy metal. The bacterial inoculum was grown on solid R2A medium at 26 °C for 3 days and suspended in distilled water to obtain a cell density of approximately OD_600_ 0.02. After incubation with Cd^2+^ for 24 h, the bacterial cells were centrifuged and rinsed three times in buffer (0.1 M Tris–HCl, 10 mM EDTA, and deionized water) to remove residual broth. The biomass of the bacterial cells was then dried and weighed. The dried bacterial pellets were digested in concentrated nitric and hydrochloric acids (1:3 *v*/*v*). The Cd^2+^ contents of the digests were analyzed using a Perkin Elmer 4100 apparatus for AAS. The influence of the Cd^2+^ concentration in the growth medium on the cell biomass and metal uptake efficiency was analyzed according to the following formula:

$$ E=\frac{\mathrm{Cd}\mathrm{A}/1000}{\mathrm{Cd}\ \mathrm{M}}\ast 100\% $$

Cd A: Amount of Cd^2+^ accumulated in the dried biomass (μg)
Cd M: Amount of Cd^2+^ in the medium (mg)

### Transmission electron microscopy/X-ray microanalysis

Elemental analysis was conducted by high-angle annular dark field scanning transmission electron microscopy (STEM-HAADF) with energy-dispersive X-ray microanalysis (EDS) for the five selected bacterial strains: *Massilia* sp. III-116-18, *Pseudomonas* sp. IV-111-14, *Bacillus* sp. ML1-2, *P. fulva*, and *S. entomophila* I-111-21. These strains exhibited the highest Cd^2+^ accumulation efficiency in the biomass (based on the results presented in Table 2). Bacterial cells were fixed and embedded in LRGold resin using a standard procedure described in Hrynkiewicz et al. (2012), with one modification: Ultrathin sections were not contrasted with osmium tetroxide (OsO_4_) to avoid osmium interference. A Tecnai F20 X-Twin electron microscope (FEI Europe), equipped with an energy dispersive X-ray (EDX) spectrometer, was used to perform qualitative and quantitative analyses of control cells and cells cultured in 2 mM Cd^2+^ to characterize the elemental composition (C, O, S, P, Na, K, Ca, Mg, and Si) and location of the Cd within the bacterial cells. The linear profile of changes in the content of Cd and other elements in the cross sections of bacterial cells was determined using the TIA software (TEM Imaging & Analysis Offline ver. 4.2 SP1, FEI Co.) based on point measurements taken every 10 nm along the diameter. These analyses were performed for six Cd-treated and three untreated (control) bacterial cells.

**Table 2:**
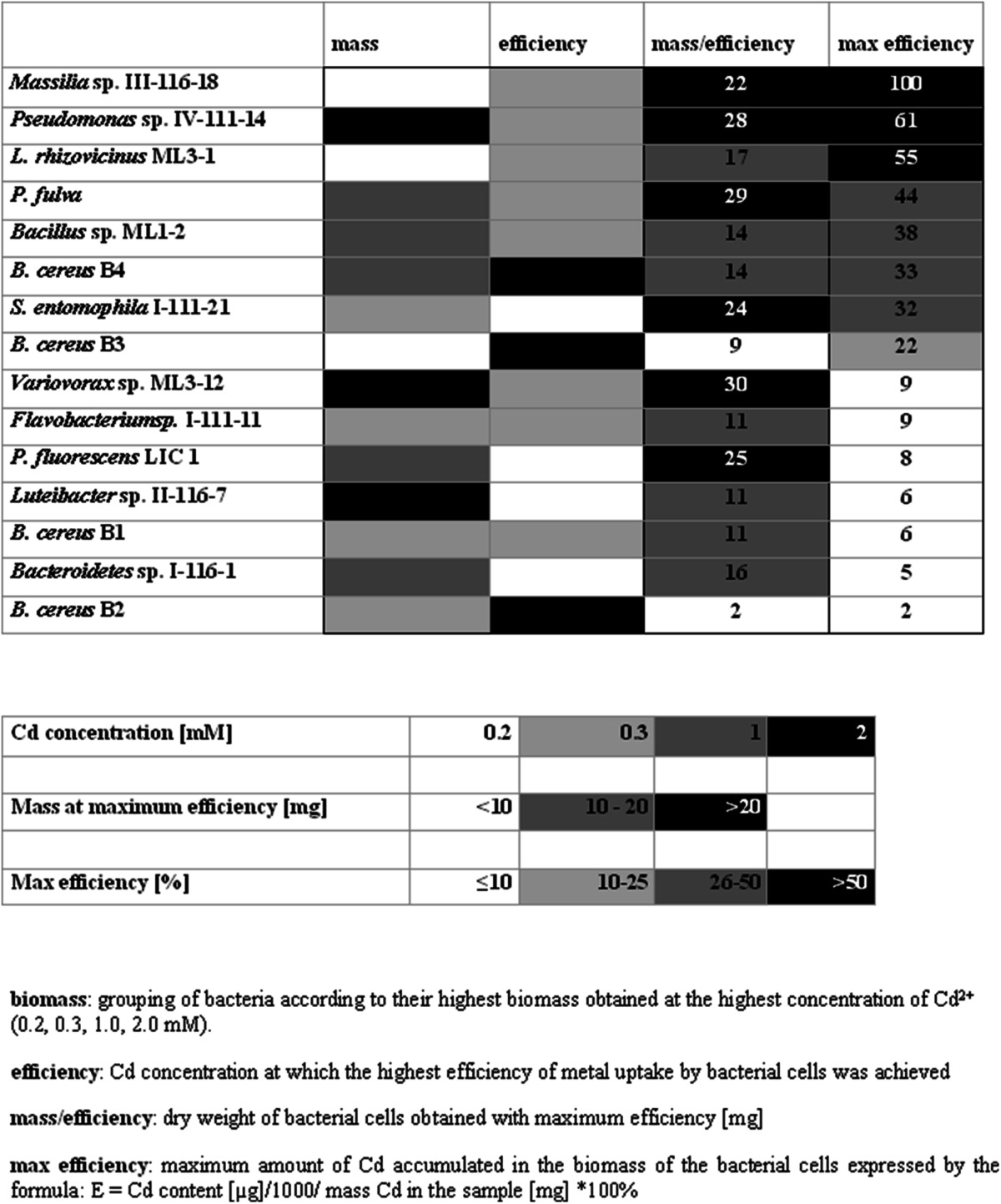
Grouping of bacteria according to biomass production in the presence of different concentrations of Cd^2+^ (0.2, 0.3, 1.0, 2.0 mM) in the medium and efficiency of bioaccumulation of Cd^2+^ based on AAS analyses

^a^Grouping of bacteria according to their highest biomass obtained at the highest concentration of Cd^2+^ (0.2, 0.3, 1.0, 2.0 mM).

^b^Cd concentration at which the highest efficiency of metal uptake by bacterial cells was achieved

^c^Dry weight of bacterial cells obtained with maximum efficiency (mg)

^d^Maximum amount of Cd accumulated in the biomass of the bacterial cells expressed by the formula E = Cd content (μg)/1,000 / mass Cd in the sample (mg) *100 %

### Statistical analyses

The differences in elemental composition between the control cells and the Cd-treated cells were analyzed by Student’s *t* test using Statistica software (Statistica ver. 7, Statsoft). The linear dependence of the Cd^2+^ content on the quantities of other elements within the bacterial cells was expressed by Pearson’s correlation coefficient, and the significance of this correlation was verified by Student’s *t* test.

## Results

The analysis of the influence of the Cd^2+^ concentration in the growth medium on the intracellular metal accumulation in the 15 studied bacterial strains revealed four classes based on biomass and three based on Cd^2+^ uptake efficiency, with no appreciable effect of bacterial origin or genus (Table 2). Three strains, *Pseudomonas* sp. IV-111-14, *Variovorax* sp. ML3-12, and *Luteibacter* sp. II-116-7, displayed the highest biomass productivity at the highest Cd^2+^ concentration (2 mM), while most of the strains (particularly *P. fulva*, *Bacillus* sp. ML1-2, *B. cereus* B4, *P. fluorescens* LIC 1, and *Bacteroidetes* sp. I-116-1) exhibited highest growth in the medium containing 1 mM Cd^2+^. In terms of Cd^2+^ uptake efficiency, three strains of *B. cereus* (B2, B3, and B4) comprised the class that exhibited the greatest metal accumulation at the highest Cd^2+^ concentration. The other 12 strains exhibited maximum Cd^2+^ accumulation capacity at 0.2 mM Cd^2+^ (*S. entomophila* I-111-21, *Variovorax* sp. ML3-12, *P. fluorescens* LIC 1, *Luteibacter* sp. II-116-7, and *Bacteroidetes* sp. I-116-1) or 0.3 mM Cd^2+^ (*Massilia* sp. III-116-18, *Pseudomonas* sp. IV-111-14, *L. rhizovicinus* ML3-1, *P. fulva*, *Bacillus* sp. ML1-2, *Flavobacterium* sp. I-111-11, and *B. cereus* B1). In the majority of cases (8 of 15 tested strains), the maximum biomass production occurred when the Cd^2+^ concentration in the medium was higher than the concentration associated with the maximum Cd^2+^ accumulation capacity. Only two strains—*B. cereus* B1 and *Flavobacterium* sp. I-111-11—exhibited the highest biomass production and Cd uptake capacity at the same Cd^2+^ concentration (0.3 mM).

Analysis of the biomass yield obtained at maximum Cd^2+^ accumulation efficiencies revealed three classes of bacterial strains (Table 2, max. efficiency). The class of bacteria characterized by a biomass production exceeding 20 mg of dry weight included six Gram-negative strains belonging to the *Gammaproteobacteria* and *Betaproteobacteria*—three *Pseudomonas* spp., *Massilia* sp. III-116-18, *S. entomophila* I-111-21, and *Variovorax* sp. ML3-12—and were mostly (four of six) isolated from rhizospheres. Fruitbody-derived *Variovorax* sp. ML3-12 reached the highest biomass growth—30 mg of dry weight (DW). The cellular biomass of seven of the bacterial strains ranged from 10 to 20 mg of DW. Only two strains of *B. cereus* (B3 and B2), which were isolated from ectomycorrhizal roots of *Salix caprea* formed by *Hebeloma* spp., produced less than 10 mg of DW at the maximum Cd^2+^ uptake capacity.

The AAS analyses revealed four groups of bacteria based on their Cd^2+^ bioaccumulation efficiency (Table 2, max. efficiency). The class characterized by the highest Cd accumulation in the bacterial cellular biomass—exceeding 50 % of the quantity of Cd^2+^ added to the medium—comprised three rhizosphere-derived Gram-negative strains (*Massilia* sp. III–116-18, *Pseudomonas* sp. IV-111-14, and *L. rhizovicinus* ML3-1). *Massilia* sp. III–116-18 exhibited the highest efficiency among these strains, taking up 100 % of the Cd^2+^ added to the medium. Four strains (*P. fulva*, *Bacillus* sp*.* ML1-2, *B. cereus* B4, and *S. entomophila* I-111-21) displayed Cd^2+^ uptake capacities of 32–44 %. The lowest efficiencies (<10 %) were observed for *Variovorax* sp. ML3-12, *Flavobacterium* sp. I-111-11, *P. fluorescens* LIC 1, *Luteibacter* sp. II-116-7, *B. cereus* B1, *Bacteroidetes* sp. I-116-1, and *B. cereus* B2; these strains were considered inefficient with respect to Cd^2+^ accumulation and were omitted from further analyses.

**Fig. 1:**
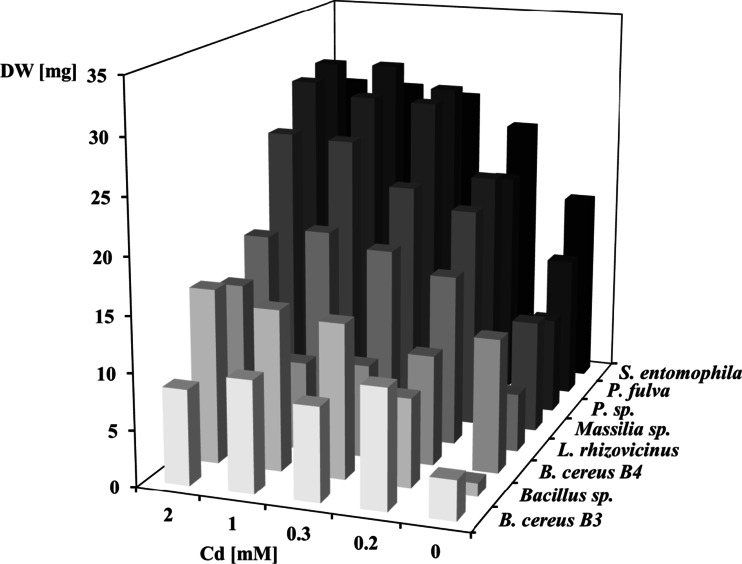
Cell biomass produced within 3 days by eight soil bacterial strains characterized by high Cd accumulation efficiency in media containing different amounts of Cd

The strains that most efficiently accumulated Cd^2+^, *Massilia* sp. III–116-18, *Pseudomonas* sp. IV-111-14, *L. rhizovicinus* ML3-1, *P. fulva*, *S. entomophila* I-111-21, and *B. cereus* B3, exhibited higher growth in the presence of Cd^2+^ compared to the control, while no significant differences in the growth of *Bacillus* sp. ML1-2 and *B. cereus* B4 were observed in the absence and presence of Cd^2+^ (Fig. 1). The biomasses of the strains *Massilia* sp. III–116-18 and *L. rhizovicinus* ML3-1 did not statistically differ with increasing Cd^2+^ content. However, *P. fulva*, *Pseudomonas* sp. IV-111-14, and *S. entomophila* I-111-21 produced significantly higher biomasses in media with Cd^2+^ concentrations ranging from 0.3 to 2.0 mM than at 0.2 mM Cd^2+^, while *B. cereus* strain B3 exhibited highest growth at the lowest Cd^2+^ concentration (0.2 mM).

**Fig. 2:**
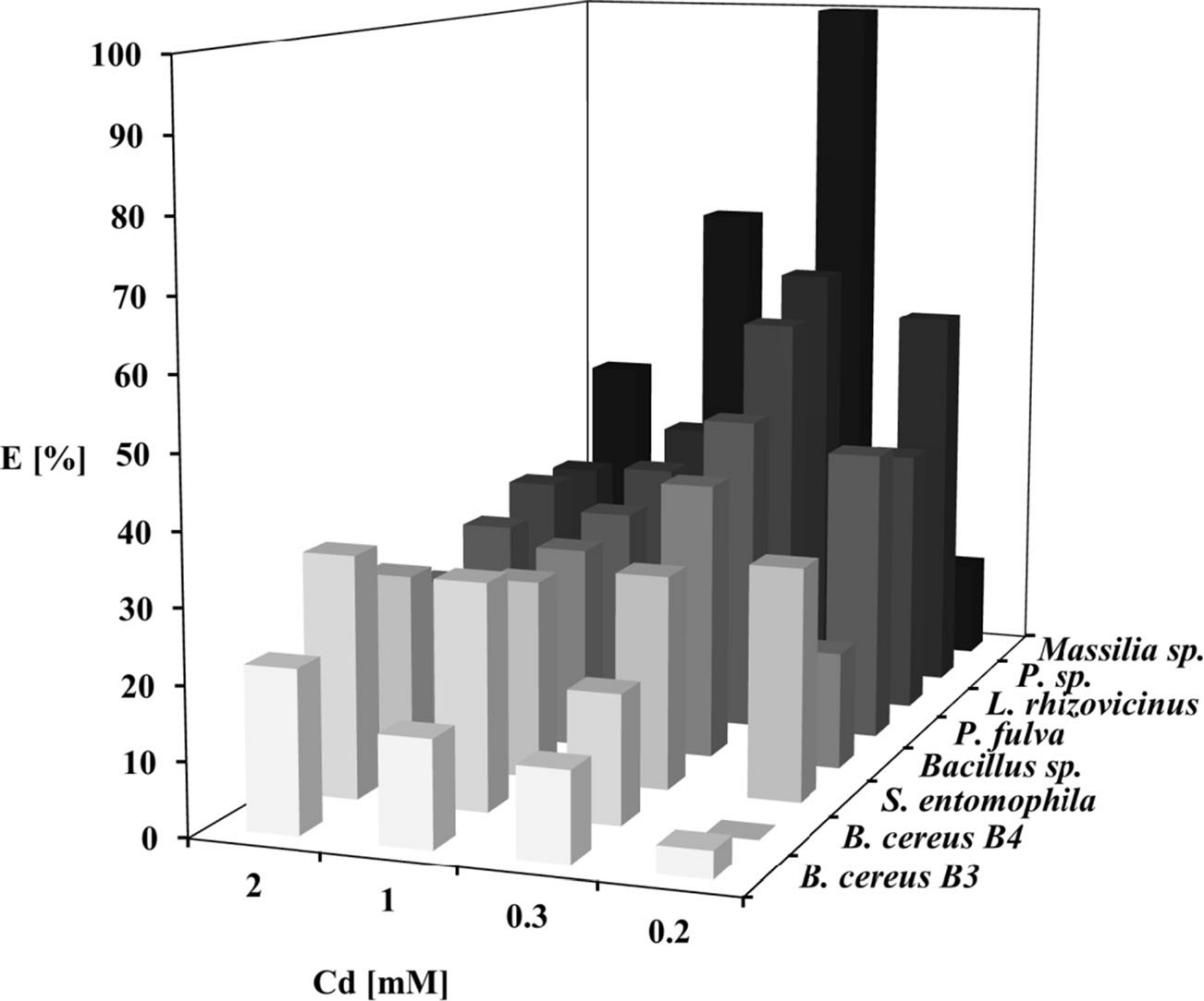
Cd accumulation capacity of eight bacterial strains characterized by high Cd uptake efficiency in culture media containing different amounts of Cd

No significant differences in the efficiency of Cd^2+^ uptake with increasing concentrations of Cd^2+^ were observed for the strain *S. entomophila* I-111-21 (Fig. 2). *B. cereus* B3 and B4 increased Cd^2+^ accumulation with increasing Cd^2+^ concentrations in the medium, while *Massilia* sp. III–116-18, *Bacillus* sp. ML1-2, and *L. rhizovicinus* ML3-1 significantly increased Cd^2+^ accumulation in 0.3 mM Cd^2+^ but gradually decreased it at higher Cd^2+^ concentrations. Two strains of *Pseudomonas* species exhibited considerably higher Cd^2+^ uptake in 0.2–0.3 mM Cd^2+^ compared to 1 and 2 mM Cd^2+^. All strains except *B. cereus* B4 produced higher biomass in the media supplemented with Cd^2+^ than in the untreated medium.

**Fig. 3:**
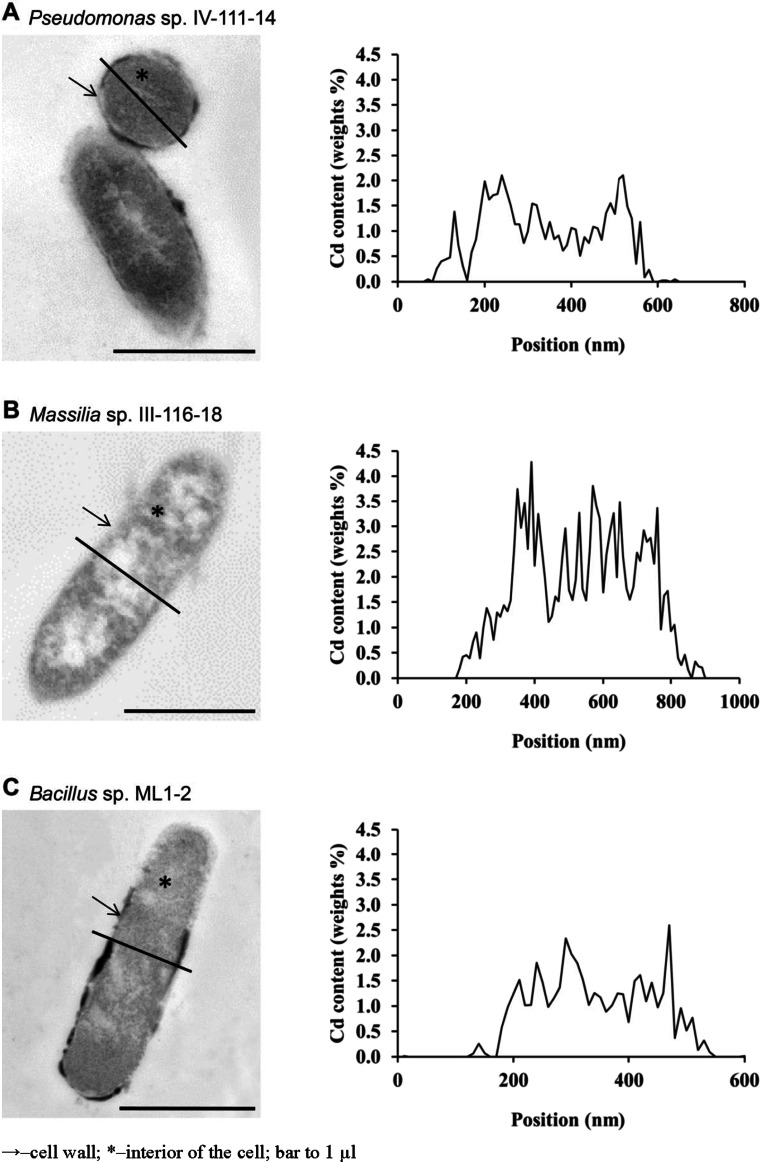
Representative STEM HAADF images indicating a linear profile of Cd content within single cells of **a** *Pseudomonas* sp. IV-111-14, **b** *Massilia* sp. III-116-18, and **c** *Bacillus* sp. ML

The Cd^2+^ and elemental nutrient composition of five highly Cd-accumulating strains were investigated in detail: *Massilia* sp. III–116-18, *Pseudomonas* sp. IV-111-14, *P. fulva*, *Bacillus* sp. ML1-2, and *S. entomophila* I-111-21. In *Massilia* sp. III–116-18, *Pseudomonas* sp. IV-111-14, and *Bacillus* sp. ML1-2, Cd was present within the cells (Tables 3–5 and Fig. 3). Intracellular Cd levels in Cd-treated *P. fulva* and *S. entomophila* I-111-21 were no different from the levels in the untreated control (Tables 6 and 7 and Fig. 3). In the cells of strains that contained substantial amounts of Cd, the metal was present both in the cell wall and the interior of the cells. The quantities of Cd differed significantly between the two investigated cellular compartments (data not shown). The quantities measured in the interior of the cells ranged from 0.87 to 1.31 weight % Cd. Cd^2+^ content was always lower in the cell wall (0.18–0.60 weight % Cd). In the strain *Massilia* sp. (which had the highest maximum Cd^2+^ accumulation efficiency according to the AAS analyses), the proportion of Cd in the cell wall was higher compared to that of the other strains (0.60 ± 0.37 weight % Cd), but the proportion of Cd in the interior of the cells did not differ from that of the other strains. A positive correlation between Cd content and K content (0.95, *p =* 0.015) inside the cells and in the cell walls of *Bacillus* sp. ML1-2 was observed. Moreover, the Cd content was correlated with the Ca content (0.94, *p = 0.*006) inside *Pseudomonas* sp. IV-111-14 cells and with the O content (0.99, *p =* 0.008) within *Massilia* sp. III–116-18 cells. In the cell walls of *Pseudomonas* sp. IV-111-14, a negative correlation of the Cd content with the K content was observed. Testing of whole cells of *Pseudomonas* sp. IV-111-14, *Massilia* sp. III–116-18, and *Bacillus* sp. ML1-2 revealed a positive correlation between the P content and Cd content in all three strains (Pearson’s *r* values: 0.89, *p =* 0.000; 0.85, *p =* 0.001; and 0.77, *p =* 0.006, respectively). Furthermore, in *Pseudomonas* sp. IV-111-14 and *Bacillus* sp. ML1-2, a significant correlation between the amounts of S and of Cd was observed. In the cells of *Massilia* sp. III–116-18 and *Pseudomonas* sp. IV-111-14, the Ca content was positively correlated with the Cd content (the linear correlation coefficients were 0.70, *p =* 0.011, and 0.95, *p =* 0.000, respectively).

**Fig. 4:**
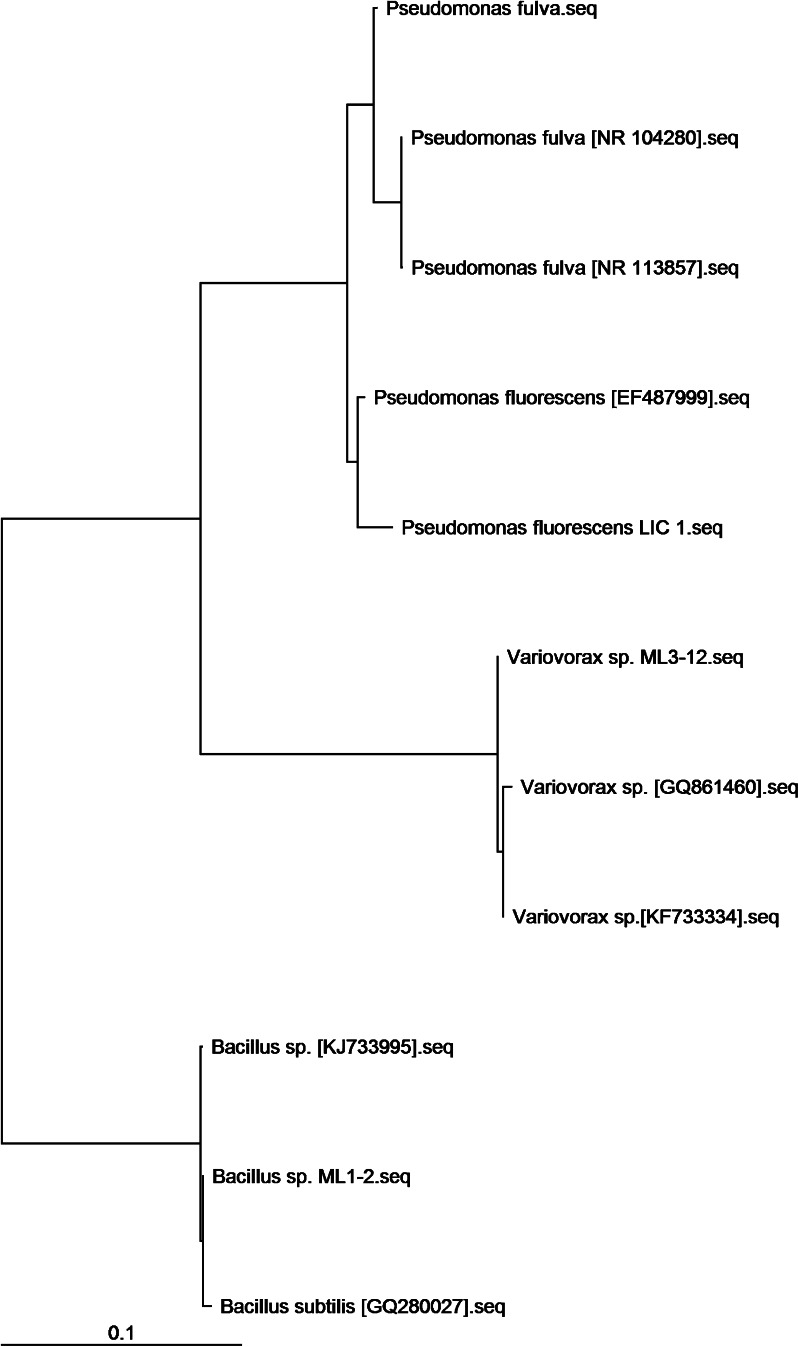
Phylogenetic relationships of newly identified bacterial strains: *Bacillus* sp. ML1-2, *Luteibacter rhizovicinus* ML3-1, *Variovorax* sp. ML3-12, and *Pseudomonas fluorescens* LIC 1 (Clustal W analysis of 16S rDNA bacterial sequences)

The analyses of the changes in elemental composition between Cd-treated and untreated cells revealed significant differences in all five studied strains. The changes in the elemental composition in response to increased Cd^2+^ concentrations in the medium were more significant inside the cells than in the cell walls. Only *Pseudomonas* sp. IV-111-14 exhibited similar decreases in P, S, K, and Ca contents in the cell wall and inside cells in response to increased Cd concentrations. The strongest changes in the elemental composition of the interior of the cells were measured in *Massilia* sp. III–116-18. In response to Cd treatment, the C content increased, and O, Mg, Si, S, P, and Ca levels decreased, while in the cell walls, only the S and Ca contents decreased. In the interior of the cells of *Bacillus* sp. ML1-2, larger changes in the elemental compositions of C, Si, P, and K were measured, while smaller changes in C and Si contents were observed in the cell walls. In the cellular compartments of *P. fulva* and *S. entomophila* I-111-21, which did not accumulate Cd^2+^ at relevant levels, the changes in elemental composition were less significant than those in the other investigated strains. However, the same number of changes (4) was observed in the interior of the cells of *S. entomophila* I-111-21 as in *Bacillus* sp. ML1-2 and *Pseudomonas* sp. IV-111-14. The whole cell analyses revealed no differences in the number of elements that changed quantities (4) between *Bacillus* sp. ML1-2 and *Pseudomonas* sp. IV-111-14 or between *Massilia* sp. III–116-18 and *P. fulva* strains. The fewest changes (2) in elemental composition were observed in *S. entomophila* I-111-21.

The Cd^2+^-mediated changes in the proportions of the individual elements in the cell wall, interior of the cells, and whole bacterial cells were investigated using a simple comparative analysis of the elemental content in Cd-treated and untreated control cells. The results were expressed as the percentage ratio of the difference between the mean values of the element quantities. The statistical outliers were rejected from the data set (Dixon’s Q test, *p* = 0.05). In addition to Cd, the analyses included elements indicated as the most abundant (C, O, and Si), light metals (Na, K, Mg, and Ca), P, and S, which may be involved in bacterial Cd^2+^ uptake. The analysis revealed two types of changes among strains that exhibited a capacity for intracellular Cd^2+^ accumulation. Increased contents of P and O were observed simultaneously with decreased amounts of C in both the cell walls and cell interior. The amounts of P and O in the interior of the cells of Cd-treated cells exceeded those in the control cells by 87–186 % and 11 %, respectively, while the C content decreased by 2–5 %. The changes in C and O contents were greater in the cell walls than in the interior of the cells and ranged from 6 to 23 % (decrease) and from 55 to 106 % (increase), respectively. Similar changes were observed in *Bacillus* sp. and *Pseudomonas* sp. strains. In addition, in *Bacillus* sp. cells, an apparent increase in Si content in the cell wall (1,298 %) and the interior of the cells (433 %) was observed. In the second type of elemental composition change, Na (30 %), Mg (48 %), and Ca (93 %) declined and K (2,293 %) increased within the interior of the cells; both decreases (Ca 92 %) and increases (Na 10 %, Si 93 %, and K 1,583 %) in the cell wall were also noted. This phenomenon was also observed for a third Cd^2+^-accumulating strain—*Massilia* sp. Changes similar to those observed in the cell wall and interior of the cells were observed in whole cells. C decreased (4–14 %) and the O and P contents increased (23–65 % and 101–118 %, respectively) in *Bacillus* sp. and *Pseudomonas* sp. in response to Cd^2+^ treatment. In addition, in whole cells of *Massilia* sp., lower P (48 %) and Ca (92 %) and higher K (1896 %) and Si (40 %) were measured in response to Cd^2+^ treatment.

## Discussion

This work is the first to analyze the bioaccumulation of Cd^2+^ ions by soil bacterial species across a broad spectrum of genera, origins, and cell structures. All 15 studied bacterial strains were able to accumulate Cd^2+^ in their cellular biomass; however, the efficiency of Cd^2+^ bioaccumulation differed significantly among the investigated strains, ranging from 2 to 100 %. These extremely dissimilar Cd^2+^ bioaccumulation capacities may be due primarily to differences in morphology, e.g., differences in the cell wall structure, which plays an important role in the biosorption of Cd^2+^ (Gourdon et al. 1990). Cell wall composition is a key factor in differentiating two basic bacterial morphotypes: Gram-negative and Gram-positive bacteria (Yun et al. 2011). Of the 15 tested strains, 10 were Gram-negative bacteria, and among these, 3 strains exhibited a maximum amount of Cd^2+^ accumulation in their biomasses (>50 %): *Massilia* sp., *Pseudomonas* sp., and *L. rhizovicinus*. The major Cd^2+^ binding sites of Gram-negative bacteria have been proposed to be located on the cell surface, which includes many carboxylate groups from the phospholipid and lipopolysaccharide characteristic of the external layer (Gourdon et al. 1990; Leung et al. 2001; Italiano et al. 2009). In the present study, *Bacillus* sp. ML1-2 and *B. cereus* B4 and B2 (22–38 %) exhibited the highest Cd^2+^ bioaccumulation efficiency among the tested Gram-positive bacteria. The lower Cd^2+^ accumulation efficiency of the Gram-positive bacteria may suggest other bioaccumulation mechanisms that are independent of passive sorption onto the cell surface. For Gram-positive strains, the process of Cd^2+^ bioadsorption can be achieved by extracellular deposition mediated by functional groups (i.e., −OH, −NH, and −PO_4_^3−^) present in the cell wall components peptidoglycan, glycoprotein, teichoic acid, and teichuronic acid (Gourdon et al. 1990; Liu et al. 2010); the last two compounds are characteristic of Gram-positive bacteria (Yee and Fein 2001). Roane et al. (2001) studied four Cd^2+^-resistant soil bacteria representing the genera *Arthrobacter*, *Bacillus*, and *Pseudomonas* and identified a plasmid-dependent intracellular mechanism of Cd^2+^ detoxification for two of these strains (*Pseudomonas* sp. H1 and *Bacillus* sp. H9). The capacities of these strains to accumulate Cd^2+^ were higher than those of strains producing an extracellular polymer layer. Based on the absence of the *cad* operon in the investigated isolates and the concurrent reduction of soluble Cd^2+^ concentrations in the medium, an intracellular mechanism of Cd^2+^ sequestration, such as metallothionein production or polyphosphate precipitation, could be assumed (Malik 2004). Roane et al. (2001) demonstrated that resistant strains belonging to the same bacterial genus may vary in their strategies of resistance to Cd^2+^, e.g., two *Pseudomonas* spp. accumulated Cd^2+^ by extracellular (*Pseudomonas* sp. 11a) or intracellular (*Pseudomonas* sp. H1) sequestration, which may indicate a strain-dependent mechanism of Cd^2+^ accumulation. This explanation was supported by Selenska–Pobell et al. (1999), who revealed that the cellular accumulation of some metal ions, including Cd^2+^, by three *Bacillus* strains (*B. cereus*, *Bacillus megaterium*, and *Bacillus sphaericus*) was species specific. Similarly, our study reveals strain-specific differences in Cd^2+^ accumulation efficiencies within the genera *Pseudomonas*, *Bacillus*, and *Luteibacter*. The differences in the Cd^2+^ uptake capacities of the strains belonging to the genera *Pseudomonas* and *Bacillus* might be due to their different origins. *Pseudomonas* sp. IV-111-14 derived from rhizosphere exhibited considerably higher Cd^2+^ accumulation efficiencies (61 %) than the *P. fluorescens* LIC1 isolated from a fruitbody (8 % accumulation of Cd^2+^). Conversely, the strains of *B. cereus* isolated from ectomycorrhizae of *Tomentella* and *Hebeloma* spp. (B1–B3) were less effective than *Bacillus* sp. ML1-2 and B4 isolated from the fruitbodies of *H. mesophaeum* (33 and 38 %, respectively). This phenomenon can be explained by a habitat-specific adaptation to high concentrations of Cd^2+^ in these strains. Plants can increase the concentration of heavy metals in the soil solution by secreting metal-mobilizing substances called phytosiderophores into the rhizosphere (Lone et al. 2008) or by secreting H^+^ ions, which can increase metal dissolution by acidification of the soil (Ali et al. 2013). Consequently, bacteria from the rhizosphere exhibited greater capacities to accumulate heavy metals than strains isolated from fruitbodies. Bacteria inhabiting ectomycorrhizal roots may be exposed to lower concentrations of Cd^2+^ due to the restriction in metal movement to roots caused by the ectomycorrhizal sheath or by binding to the cell wall of the mycelium (Hall 2002).

The most effective strains for Cd^2+^ accumulation belonged to the genera *Pseudomonas* (Selenska-Pobell et al. 2001; Pardo et al. 2003; Ziagova et al. 2007; Choudhary and Sar 2009) and *Bacillus* (Selenska-Pobell et al. 2001; Zouboulis et al. 2004; Yilmaz and Ensari 2005); high Cd^2+^ accumulation by species of these genera has been previously reported. However, this study is the first to report Cd^2+^ accumulation in a *Massilia* sp. strain*. Massilia* sp. III–116-18 exhibited the highest Cd^2+^ uptake at Cd^2+^ concentrations of 0.3–2 mM. The high potential for Cd^2+^ uptake observed for strains of *L. rhizovicinus* and *S. entomophila* in the present study has not been extensively reported. In one of the few papers describing this aspect of *Serratia* sp., Choudhary et al. (2012) reported high Cd^2+^ resistance and accumulation capacities of the tested strain. The present results suggest that an extension of the screening of bacterial taxa for Cd^2+^ bioaccumulation to improve the efficiency of bioremediation of contaminated soils or sludge would be promising because only a few taxa have been evaluated in detail and many highly valuable candidates might still be unknown.

In our work, the majority of the tested bacterial strains (*Massilia* sp. III–116-18, *Pseudomonas* sp. IV-111-14, *L. rhizovicinus* ML3-1, *P. fulva*, *S. entomophila* I-111-21, *B. cereus* B3, *B. cereus* B4, and *Bacillus* sp. ML1-2) did not exhibit significant changes in biomass or actually increased biomass production when grown in media containing increasing concentrations of Cd^2+^. This phenomenon was discussed by Roane and Pepper (2000), who observed higher biomass production by *Pseudomonas* H1 at Cd^2+^ concentrations of 60, 150, and 300 μg ml^−1^ compared to lower Cd^2+^ concentrations (48 μg Cd ml^−1^). The authors suggested that the increased resistance of bacteria with increasing Cd^2+^ concentrations might be due to a shift in the mechanism of resistance under more stressful conditions. Naz et al. (2005) did not observed any changes in the growth of five investigated sulfate-reducing bacteria in the presence of subtoxic Cd^2+^ concentrations compared to the control without toxic ions.

Microscopic analysis of the Cd distribution within the cells of the five most efficient Cd-accumulating bacterial strains revealed intracellular accumulation by *Massilia* sp. III–116-18, *Pseudomonas* sp. IV-111-14, and *Bacillus* sp. ML1-2. Cd^2+^ was bound in both the cell wall and within the interior of the cells, and the amounts measured in the cytosol were significantly higher compared to those measured in the peripheral parts of the cells. This finding may suggest the presence of Cd-binding and/or efflux mechanisms inside the cells that mediate resistance against Cd^2+^ toxicity. Similar to our findings, Trevors et al. (1986) demonstrated intracellular Cd^2+^ sequestration in a cysteine-rich-protein-producing strain of *P. putida* isolated from sewage. Both periplasmic and cytoplasmic sequestrations of Cd^2+^ in *Escherichia coli* due to metallothionein expression were observed by Pazirandeh et al. (1998) and Yoshida et al. (2002). Similarly, the simultaneous accumulation of Cd^2+^ in the cell envelope and within the cytoplasm was reported by Choudhary and Sar (2009) and Sinha and Mukherjee (2009); however, the investigated *Pseudomonas* strains preferentially sequestrated Cd^2+^ in and around the cell periphery compared to the cytoplasm.

In the present study, the absence of Cd^2+^ within the cells of *P. fulva* and *S. entomophila* I-111-21 suggests cell surface binding of Cd^2+^ as a dominant detoxification mechanism. Siripornadulsil and Siripornadulsil (2013) investigated the Cd^2+^ accumulation ability of 24 Gram-negative bacteria isolated from rice fields and observed cell-surface- or cell-wall-bound Cd^2+^, which was easily washed out by EDTA. A similar phenomenon was reported for vegetative cells of *Bacillus* strains isolated from the drain waters of a uranium waste dump, where most of the heavy metals accumulated were easily extracted with EDTA/Tris solution (Selenska-Pobell et al. 1999). Thus, we suggest that the absence of Cd peaks in energy dispersive X-ray spectra for both *P. fulva* and *S. entomophila* I-111-21 cells in the present study could be the result of chelation and subsequent rinsing of the surface-bound Cd^2+^.

Based on the observed differences in elemental composition within the cell walls and interior of the cells between Cd^2+^-treated and control cells of the investigated bacterial strains, we suggest three different mechanisms of intracellular Cd^2+^-accumulation. The first mechanism is related to the increased accumulation of phosphate detected in *Pseudomonas* sp. IV-111-16 and *Bacillus* sp. ML1-2. Because cadmium phosphate is poorly soluble (Aiking et al. 1984) and significantly increased levels of phosphate were observed in the Cd^2+^-treated bacterial cells (compared to bacterial cells cultivated without Cd^2+^), this phenomenon might indicate a mechanism of detoxification by intracellular sequestration. This suggestion is supported by previous reports (Aiking et al. 1984; Keasling and Hupf 1996) indicating a clear correlation between increased resistance to Cd^2+^ and increased phosphate content inside *K. aerogenes* and *E. coli* cells. Moreover, some authors suggest that polyphosphate granules present in bacterial cells are responsible for the intracellular binding of metals (Volesky et al. 1992; Volesky and May-Philips 1995). The second mechanism assumes that some Gram-positive bacteria (e.g., *Bacillus subtilis*) are able to retain several heavy metals as silicate minerals (Mera and Beveridge 1993a). This mechanism could explain the results observed for the *Bacillus* sp. ML1-2 strain analyzed in our work. The binding of silicate and the formation of metallic salts on the surface of Gram-positive bacteria cells could occur in two different ways: (i) by the binding of SiO_3_^2−^ to positively charged groups present in the teichoic acids and in the peptidoglycan or (ii) by binding of SiO_3_^2−^ through cationic bridging by wall-bound metallic cations (Mera and Beveridge 1993b). However, the *Bacillus* sp. ML1-2 strain investigated in this study exhibited an increased content of silicates within the interior of the cells, in contrast to the proposed definition. A third mechanism, ion exchange, has been suggested as a leading process for Cd^2+^ entry into cells for *Massilia* sp. III-116-18. For this bacterial strain, significant changes in the amounts of Na, Mg, Ca, and K in the interior of the cells and Na, Ca, and K in the cell wall were observed. The replacement of Ca^2+^ by Cd^2+^ in the cell wall could be explained by the ion exchange properties of polysaccharides, in which metallic ions can exchange with the counter ions such as Zn^2+^, Cu^2+^, or Cd^2+^ (based on the hard-and-soft principle proposed by Avery and Tobin (1993)), resulting in their accumulation (Veglio and Beolchini 1997). A simultaneous increase in Na and K content may indicate cation competition between monovalent cations and Cd^2+^, which has been previously demonstrated for Na^+^ and Tl^+^ in *Saccharomyces cerevisiae* (Avery and Tobin 1993), but this phenomenon is not clear. The elemental changes within the interior of *Massilia* sp. III-116-18 cells indicated a phenomenon of Ca and Mg release from bacteria during metal accumulation (Choudhary and Sar 2009). However, the increased intracellular K content observed in our studies is in contrast to the findings of Choudhary and Sar (2009), who used the same EDX technique and observed a significant loss of cellular K after metal uptake by metal-resistant *Pseudomonas* sp. isolated from a uranium mine (from 25.7 to 3.5 % and less). However, in a study by Nies (1992) similar to our study, a stimulatory effect of Cd^2+^ on K^+^ uptake was observed in *R. metallidurans* CH34. Thus, referring to the claim of Guffanti et al. (2002), K^+^ uptake may have a protective function in conjunction with efflux activity.

## Conclusions

The ability of soil bacteria to accumulate Cd^2+^ is strain specific, and the variation within a species can exceed the variations between different species or genera. The rhizosphere appears to be a promising source of highly Cd-tolerant and Cd-accumulating bacterial strains. The mechanisms of Cd^2+^ tolerance vary by strain and cause a strain-specific pattern of response to increased Cd^2+^ concentrations in the medium. Within bacterial cells, Cd^2+^ is mainly accumulated in the interior of the cells and in smaller quantities in the cell walls. The present results suggest that an extension of the screening of bacterial taxa for Cd^2+^ bioaccumulation to improve the efficiency of bioremediation of contaminated soils might yield promising results because only a few taxa have been evaluated in detail and many highly valuable candidates might be still unknown. As the most promising, we suggest strains from the genus *Massilia* sp., *Pseudomonas* sp., and *Bacillus* sp.

## Electronic supplementary material

Below is the link to the electronic supplementary material.

ESM 1 (DOCX 37 kb)
