# Supplementary material for: Strain-specific bioaccumulation and intracellular distribution of Cd2+ in bacteria isolated from the rhizosphere, ectomycorrhizae, and fruitbodies of ectomycorrhizal fungi
Source: Environ Sci Pollut Res Int. 2014 Sep 19;22(4):3055–67. doi: 10.1007/s11356-014-3489-0 (PMC4315882; doi:10.1007/s11356-014-3489-0)
Supplement: Supplementary file 1 — (DOCX 37 kb) [file 11356_2014_3489_MOESM1_ESM.docx]

**Table 3** Comparison of the elemental composition of Cd-treated and untreated *Bacillus* sp. ML1-2 cells. Values are expressed as the mean and standard deviation of the percentage of the atomic weights of the individual elements.

| Part | Variant | **C(K)** | **O(K)** | **Na(K)** | **Mg(K)** | **Si(K)** | **P(K)** | **S(K)** | **K(K)** | **Ca(K)** | **Cd(L)** |
| --- | --- | --- | --- | --- | --- | --- | --- | --- | --- | --- | --- |
| IC | Ctr | 91.25 ± 1.10 | 7.09 ± 0.63 | 0.14 ± 0.02 | 0.08 ± 0.02 | 0.40 ± 0.06 | 0.58 ± 0.23 | 0.14 ± 0.06 | 0.01 ± 0.00 | 0.26 ± 0.14 | 0.02 ± 0.01 |
|  | Cd | 86.56 ± 1.60↓↓ | 7.86 ± 1.35 | 0.11 ± 0.02 | 0.08 ± 0.01 | 2.13 ± 0.61↑↑ | 1.65 ± 0.33↑↑ | 0.17 ± 0.07 | 0.05 ± 0.02↑↑ | 0.30 ± 0.06 | 1.19 ± 0.25↑↑ |
| CW | Ctr | 90.14 ± 2.39 | 7.28 ± 1.10 | 0.13 ± 0.03 | 0.08 ± 0.02 | 1.11 ± 1.09 | 0.78 ± 0.26 | 0.10 ± 0.04 | 0.01 ± 0.00 | 0.33 ± 0.18 | 0.02 ± 0.01 |
|  | Cd | 69.75 ± 10.16↓ | 14.99 ± 3.77 | 0.11 ± 0.02 | 0.07 ± 0.03 | 15.46 ± 5.68↑↑ | 1.07 ± 0.54 | 0.09 ± 0.03 | 0.02 ± 0.01 | 0.21 ± 0.26 | 0.26 ± 0.23 |
| Cell | Ctr | 91.32 ± 1.01 | 6.93 ± 0.13 | 0.13 ± 0.02 | 0.08 ± 0.02 | 0.43 ± 0.07 | 0.68 ± 0.25 | 0.12 ± 0.05 | 0.01 ± 0.00 | 0.30 ± 0.15 | 0.02 ± 0.01 |
|  | Cd | 78.16 ± 11.19↓ | 11.42 ± 4.60 | 0.11 ± 0.02↓ | 0.07 ± 0.02 | 7.75 ± 7.63 | 1.36 ± 0.52↑↑ | 0.13 ± 0.06 | 0.03 ± 0.02↑↑ | 0.25 ± 0.19 | 0.73 ± 0.49↑↑ |
| Cd corr. | Cell | **0.71/0.015*** | **-0.74/0.010** | -0.06/0.864 | 0.52/0.101 | **-0.74/0.010** | **0.77/0.006** | **0.88/0.000** | **0.93/0.000** | 0.33/0.316 |  |
|  | IC | -0.75/0.146 | 0.72/0.175 | 0.53/0.359 | 0.72/0.175 | -0.43/0.468 | 0.77/0.132 | 0.81/0.097 | **0.95/0.015** | 0.82/0.086 |  |
|  | CW | 0.75/0.084 | -0.73/0.101 | -0.19/0.720 | 0.58/0.229 | -0.85/0.072 | 0.73/0.099 | 0.65/0.159 | 0.79/0.061 | 0.76/0.078 |  |

Abbreviations:

↑–significantly higher compared to the control at p level < 0.05; ↑↑–significantly higher compared to the control at p level < 0.01

↓–significantly lower compared to the control at p level < 0.05; ↓↓–significantly lower compared to the control at p level < 0.01

*– Pearson's *r* / p level

Ctr: control medium without Cd treatment; Cell: total cell; IC: interior of the bacterial cell; CW: cell wall

**Table 4** Comparison of the elemental composition of Cd-treated and untreated *Massilia* sp. III-116-18 cells. The values are expressed as the mean and standard deviation of the percentage of the atomic weights of the individual elements.

| Part | Variant | **C(K)** | **O(K)** | **Na(K)** | **Mg(K)** | **Si(K)** | **P(K)** | **S(K)** | **K(K)** | **Ca(K)** | **Cd(L)** |
| --- | --- | --- | --- | --- | --- | --- | --- | --- | --- | --- | --- |
| IC | C | 83.62 ± 0.32 | 9.49 ± 0.32 | 0.57 ± 0.06 | 0.18 ± 0.03 | 2.34 ± 0.38 | 2.49 ± 0.68 | 0.32 ± 0.04 | 0.01 ± 0.00 | 0.93 ± 0.15 | 0.01 ± 0.00 |
|  | Cd | 87.96 ± 0.95↑↑ | 7.44 ± 0.74↓↓ | 0.40 ± 0.32 | 0.09 ± 0.03↓↓ | 1.65 ± 0.21↓↓ | 1.10 ± 0.16↓↓ | 0.14 ± 0.03↓↓ | 0.13 ± 0.08 | 0.07 ± 0.02↓↓ | 1.31 ± 0.43↑ |
| CW | C | 86.66 ± 2.78 | 7.86 ± 1.49 | 0.35 ± 0.12 | 0.10 ± 0.04 | 3.06 ± 0.29 | 1.13 ± 0.52 | 0.22 ± 0.08 | 0.01 ± 0.01 | 0.59 ± 0.31 | 0.00 ± 0.00 |
|  | Cd | 80.53 ± 7.44 | 9.99 ± 3.06 | 0.38 ± 0.31 | 0.08 ± 0.02 | 5.89 ± 4.87 | 0.74 ± 0.11 | 0.11 ± 0.03↓ | 0.10 ± 0.08 | 0.05 ± 0.02↓↓ | 0.60 ± 0.37↑ |
| Cell | C | 85.14 ± 2.43 | 9.17 ± 0.61 | 0.46 ± 0.15 | 0.14 ± 0.06 | 2.70 ± 0.49 | 1.81 ± 0.92 | 0.27 ± 0.08 | 0.01 ± 0.01 | 0.76 ± 0.29 | 0.01 ± 0.01 |
|  | Cd | 84.61 ± 5.95 | 8.72 ± 2.51 | 0.53 ± 0.47 | 0.09 ± 0.03↓ | 3.77 ± 3.96 | 0.94 ± 0.23↓ | 0.15 ± 0.09↓ | 0.11 ± 0.08↑ | 0.06 ± 0.02↓↓ | 0.98 ± 0.64↑↑ |
| Cd corr. | Cell | 0.42/0.176* | -0.47/0.121 | 0.26/0.409 | 0.45/0.142 | **-0.63/0.028** | **0.85/0.001** | **0.77/0.003** | -0.34/0.300 | **0.70/0.011** |  |
|  | IC | -0.81/0.189 | **0.99/0.008** | -0.81/0.186 | -0.94/0.057 | -0.93/0.068 | 0.36/0.641 | 0.20/0.798 | -0.83/0.172 | 0.58/0.416 |  |
|  | CW | **0.87/0.024** | **-0.84/0.037** | -0.46/0.356 | 0.29/0.574 | **-0.89/0.018** | 0.77/0.128 | 0.32/0.539 | -0.29/0.574 | 0.61/0.197 |  |

**Abbreviations:**Abbreviations: Same as in Table 3

**Table 5** Comparison of the elemental composition of Cd-treated and untreated *Pseudomonas* sp. IV- 111-14 cells. The values are expressed as the mean and standard deviation of the percentage of the atomic weights of the individual elements.

| Part | Variant | **C(K)** | **O(K)** | **Na(K)** | **Mg(K)** | **Si(K)** | **P(K)** | **S(K)** | **K(K)** | **Ca(K)** | **Cd(L)** |
| --- | --- | --- | --- | --- | --- | --- | --- | --- | --- | --- | --- |
| IC | C | 85.38 ± 0.87 | 8.72 ± 0.59 | 0.12 ± 0.01 | 0.07 ± 0.02 | 4.27 ± 0.14 | 0.99 ± 0.12 | 0.12 ± 0.02 | 0.01 ± 0.00 | 0.26 ± 0.05 | 0.02 ± 0.01 |
|  | Cd | 84.10 ± 2.33 | 9.69 ± 1.86 | 0.10 ± 0.03 | 0.09 ± 0.03 | 3.32 ± 0.65 | 1.84 ± 0.23↑↑ | 0.22 ± 0.07↑ | 0.03 ± 0.01↑ | 0.46 ± 0.14↑ | 0.87 ± 0.34↑↑ |
| CW | C | 86.66 ± 0.67 | 7.63 ± 0.37 | 0.06 ± 0.02 | 0.04 ± 0.01 | 5.28 ± 0.23 | 0.20 ± 0.04 | 0.03 ± 0.02 | 0.00 ± 0.00 | 0.05 ± 0.01 | 0.01 ± 0.00 |
|  | Cd | 81.44 ± 4.45 | 11.81 ± 3.91 | 0.09 ± 0.04 | 0.06 ± 0.02↑ | 6.75 ± 2.07 | 0.75 ± 0.26↑ | 0.10 ± 0.04↑ | 0.02 ± 0.01↑ | 0.20 ± 0.06↑↑ | 0.18 ± 0.08↑↑ |
| Cell | C | 86.02 ± 0.98 | 8.17 ± 0.74 | 0.09 ± 0.04 | 0.06 ± 0.02 | 4.78 ± 0.58 | 0.60 ± 0.44 | 0.08 ± 0.05 | 0.01 ± 0.00 | 0.15 ± 0.12 | 0.02 ± 0.01 |
|  | Cd | 82.20 ±3.93 | 10.04 ± 2.01 | 0.09 ± 0.03 | 0.08 ± 0.03 | 5.15 ± 2.23 | 1.30 ± 0.62↑ | 0.16 ± 0.08↑ | 0.03 ± 0.01↑↑ | 0.33 ± 0.17↑ | 0.56 ± 0.43↑ |
| Cd corr. | Cell | -0.24/0.503* | 0.24/0.500 | 0.37/0.265 | **0.71/0.015** | -0.33/0.349 | **0.89/0.000** | **0.88/0.000** | **0.76/0.007** | **0.95/0.000** |  |
|  | IC | -0.77/0.125 | 0.75/0.085 | 0.34/0.513 | 0.53/0.281 | 0.51/0.384 | 0.71/0.117 | 0.78/0.066 | 0.71/0.116 | **0.94/0.006** |  |
|  | CW | -0.61/0.272 | 0.77/0.071 | 0.44/0.388 | -0.61/0.203 | 0.66/0.228 | 0.81/0.051 | 0.27/0.600 | **-0.91/0.013** | 0.36/0.486 |  |

Abbreviations: Same as in Table 3

**Table 6** Comparison of the elemental composition of Cd-treated and untreated *Pseudomonas fulva* cells. The values are expressed as the mean and standard deviation of the percentage of the atomic weights of the individual elements.

| Part | Variant | **C(K)** | **O(K)** | **Na(K)** | **Mg(K)** | **Si(K)** | **P(K)** | **S(K)** | **K(K)** | **Ca(K)** | **Cd(L)** |
| --- | --- | --- | --- | --- | --- | --- | --- | --- | --- | --- | --- |
| IC | C | 88.41 ± 1.83 | 6.38 ± 1.23 | 0.13 ± 0.01 | 0.06 ± 0.01 | 2.85 ± 1.07 | 0.95 ± 0.18 | 0.32 ± 0.13 | 0.01 ± 0.00 | 0.84 ± 0.79 | 0.02 ± 0.01 |
|  | Cd | 91.42 ± 1.78↑ | 5.25 ± 1.16 | 0.25 ± 0.15 | 0.09 ± 0.03 | 0.83 ± 0.10↓↓ | 1.55 ± 0.23↑ | 0.18 ± 0.06 | 0.16 ± 0.21 | 0.25 ± 0.09 | 0.09 ± 0.04 |
| CW | C | 85.74 ± 2.43 | 7.52 ± 1.47 | 0.11 ± 0.03 | 0.05 ± 0.00 | 5.03 ± 0.86 | 0.55 ± 0.05 | 0.24 ± 0.20 | 0.01 ± 0.01 | 0.69 ± 0.85 | 0.01 ± 0.01 |
|  | Cd | 93.35 ± 1.19↑↑ | 4.39 ± 0.85↓↓ | 0.15 ± 0.07 | 0.06 ± 0.02 | 0.84 ± 0.07↓↓ | 0.81 ± 0.21 | 0.11 ± 0.04 | 0.09 ± 0.09 | 0.20 ± 0.09 | 0.03 ± 0.02 |
| Cell | C | 86.43 ± 2.04 | 6.95 ± 1.37 | 0.12 ± 0.03 | 0.06 ± 0.01 | 4.78 ± 0.86 | 0.75 ± 0.25 | 0.28 ± 0.16 | 0.01 ± 0.01 | 0.77 ± 0.74 | 0.01 ± 0.01 |
|  | Cd | 92.38 ± 1.76↑↑ | 4.82 ± 1.07↓↓ | 0.17 ± 0.07 | 0.08 ± 0.03 | 0.84 ±0.08↓↓ | 1.05 ± 0.50 | 0.15 ± 0.06↓ | 0.08 ± 0.07 | 0.22 ± 0.09↓ | 0.07 ± 0.04 |
| Cd corr. | Cell | **-0.72/0.008*** | **0.78/0.003** | 0.14/0.655 | **0.74/0.006** | -0.08/0.797 | 0.52/0.082 | 0.31/0.331 | -0.15/0.634 | **0.80/0.002** |  |
|  | IC | -0.35/0.492 | 0.61/0.202 | -0.32/0.538 | 0.68/0.134 | -0.53/0.282 | -0.08/0.885 | -0.07/0.902 | -0.47/0.344 | 0.74/0.095 |  |
|  | CW | **-0.97/0.001** | **0.95/0.003** | 0.21/0.691 | 0.55/0.258 | 0.78/0.068 | 0.73/0.102 | -0.08/0.876 | -0.25/0.628 | 0.80/0.058 |  |

Abbreviations: Same as in Table 3

**Table 7** Comparison of the elemental composition of Cd-treated and untreated *Serratia entomophila* I-111-21 cells. The values are expressed as the mean and standard deviation of the percentage of the atomic weights of the individual elements.

| Part | Variant | **C(K)** | **O(K)** | **Na(K)** | **Mg(K)** | **Si(K)** | **P(K)** | **S(K)** | **K(K)** | **Ca(K)** | **Cd(L)** |
| --- | --- | --- | --- | --- | --- | --- | --- | --- | --- | --- | --- |
| IC | C | 87.79 ± 1.10 | 8.40 ± 0.76 | 0.83 ± 0.11 | 0.42 ± 0.06 | 1.06 ± 0.02 | 0.60 ± 0.04 | 0.42 ± 0.06 | 0.10 ± 0.01 | 0.28 ± 0.05 | 0.05 ± 0.01 |
|  | Cd | 86.57 ± 0.36 | 7.80 ± 0.36 | 0.53 ± 0.13 | 0.32± 0.06 | 2.17 ± 0.17↑↑ | 1.11 ± 0.07↑↑ | 0.47 ± 0.17 | 0.02 ± 0.01↓↓ | 1.18 ± 0.24↑↑ | 0.03 ± 0.00 |
| CW | C | 66.23 ± 0.42 | 16.43 ± 1.20 | 0.41 ± 0.05 | 0.12 ± 0.01 | 16.01 ± 1.68 | 0.32 ± 0.06 | 0.18 ± 0.02 | 0.05 ± 0.00 | 0.19 ± 0.04 | 0.02 ± 0.02 |
|  | Cd | 82.10 ± 7.09 | 10.29 ± 3.25 | 0.28 ± 0.07 | 0.14 ± 0.04 | 5.88 ± 3.17 | 0.51 ± 0.24 | 0.32 ± 0.35 | 0.02 ± 0.01↓↓ | 0.49 ± 0.24 | 0.03 ± 0.01 |
| Cell | C | 77.01 ± 12.47 | 12.41 ± 4.71 | 0.62 ± 0.25 | 0.27 ± 0.18 | 8.54 ± 8.69 | 0.46 ± 0.17 | 0.30 ± 0.14 | 0.07 ± 0.03 | 0.24 ± 0.07 | 0.04 ± 0.03 |
|  | Cd | 84.78 ± 5.54 | 8.87 ± 2.65 | 0.37 ± 0.18↓ | 0.21 ± 0.12 | 3.85 ± 2.79↓ | 0.73 ± 0.40 | 0.36 ± 0.30 | 0.02 ± 0.01↓↓ | 0.74 ± 0.47 | 0.03 ± 0.01 |
| Cd corr. | Cell | **-0.81/0.001*** | **0.81/0.001** | 0.41/0.187 | 0.29/0.365 | **0.69/0.012** | 0.50/0.095 | 0.30/0.348 | 0.48/0.113 | 0.18/0.568 |  |
|  | IC | **-0.83/0.043** | 0.78/0.066 | 0.80/0.056 | 0.78/0.068 | 0.17/0.753 | **0.92/0.009** | 0.67/0.148 | 0.73/0.103 | **0.81/0.049** |  |
|  | CW | **-0.88/0.019** | **0.91/0.012** | 0.80/0.054 | 0.58/0.224 | **0.88/0.020** | **0.85/0.032** | 0.19/0.725 | 0.42/0.403 | -0.11/0.838 |  |

Abbreviations: Same as in Table 3

**Figure 4**
